# Updated Systematic Review and Meta-Analysis of the Performance of Risk Prediction Rules in Children and Young People with Febrile Neutropenia

**DOI:** 10.1371/journal.pone.0038300

**Published:** 2012-05-31

**Authors:** Robert S. Phillips, Thomas Lehrnbecher, Sarah Alexander, Lillian Sung

**Affiliations:** 1 Centre for Reviews and Dissemination, University of York, York, United Kingdom; 2 Department of Paediatric Haematology and Oncology, University of Frankfurt, Frankfurt, Germany; 3 Division of Haematology/Oncology, The Hospital for Sick Children, Toronto, Canada; Aga Khan University, Pakistan

## Abstract

**Introduction:**

Febrile neutropenia is a common and potentially life-threatening complication of treatment for childhood cancer, which has increasingly been subject to targeted treatment based on clinical risk stratification. Our previous meta-analysis demonstrated 16 rules had been described and 2 of them subject to validation in more than one study. We aimed to advance our knowledge of evidence on the discriminatory ability and predictive accuracy of such risk stratification clinical decision rules (CDR) for children and young people with cancer by updating our systematic review.

**Methods:**

The review was conducted in accordance with Centre for Reviews and Dissemination methods, searching multiple electronic databases, using two independent reviewers, formal critical appraisal with QUADAS and meta-analysis with random effects models where appropriate. It was registered with PROSPERO: CRD42011001685.

**Results:**

We found 9 new publications describing a further 7 new CDR, and validations of 7 rules. Six CDR have now been subject to testing across more than two data sets. Most validations demonstrated the rule to be less efficient than when initially proposed; geographical differences appeared to be one explanation for this.

**Conclusion:**

The use of clinical decision rules will require local validation before widespread use. Considerable uncertainty remains over the most effective rule to use in each population, and an ongoing individual-patient-data meta-analysis should develop and test a more reliable CDR to improve stratification and optimise therapy. Despite current challenges, we believe it will be possible to define an internationally effective CDR to harmonise the treatment of children with febrile neutropenia.

## Introduction

Febrile neutropenia (FNP) is a common and potentially life-threatening complication of therapy for childhood cancer, which has increasingly been subject to targeted treatment based on clinical risk stratification [1]. For children this move towards risk-directed care is based upon evidence of the low incidence of death [2], the majority of patients being without identified significant infection or sepsis [3], and small randomised trials demonstrating the feasibility of out-patient based treatment for patients at low-risk of septic complications [4]. A large proportion of the evidence for risk stratifications has originated from adult oncology [5] It is acknowledged that children are not ‘little adults’ but distinct in the biology of their malignancies, treatment regimens, infections and psychosocial setting and therefore specific evidence for stratification of children with FNP is needed [6].

Since we undertook a systematic review and meta-analysis of risk stratification systems in 2008 [3], further studies have been published which address this issue [7]. Accordingly we have updated our review to summarise the most recent advances in our knowledge of evidence on the discriminatory ability and predictive accuracy of such risk stratification clinical decision rules (CDR) for children and young people with cancer.

## Methods

This update review was conducted in accordance with “Systematic reviews: CRD's guidance for undertaking reviews in health care” [8] and registered on the PROSPERO Registry of systematic reviews: CRD42011001685. It sought studies which aimed to derive or validate a CDR in children or young people (aged 0–18 y) presenting with febrile neutropenia. Both prospective and retrospective cohorts were included, but those using a case-control (“two-gate”) approach were excluded as these have been previously shown to exaggerate diagnostic accuracy estimates [9].

### Search strategy and selection criteria

The electronic search strategy [3] was reviewed and repeated on the following databases from February 2009 to September 2011:

- MEDLINE
- MEDLINE In-Process & Other Non-Indexed Citations
- EMBASE
- CINAHL
- Literatura Latinoamericana y del Caribe en Ciencias de la Salud (LILACS)

Reference lists of relevant systematic reviews and included articles were reviewed for further relevant articles. Published and unpublished studies were sought and no language restrictions applied. Non-English language studies were translated. Two reviewers independently screened the title and abstract of studies for inclusion, and then the full text of retrieved articles. Disagreements were resolved by consensus.

### Validity assessment and data extraction

The validity of each study was assessed as with our previous review using 11 of the 14 questions from the QUADAS assessment tool for diagnostic accuracy studies [10].

Data were extracted by one reviewer and checked by the other. The data extracted included age and sex distribution of the included participants, geographical location of the study, the participant inclusion/exclusion criteria, and the performance of the CDR as a 2*k table (where *k* refers to the number of strata described) or as sensitivity/specificity, as well as aspects of the methods used to derive the CDR (where applicable).

### Methods of analysis/synthesis

Where possible, data from new publications were added to meta-analyses undertaken in the original review [3]. Quantitative synthesis was undertaken when more than 2 studies tested the same CDR, and where appropriate, was investigated for sources for heterogeneity. For this update review, only dichotomous test data were found. For CDR with 3 datasets, a univariate approach was used (pooling sensitivity and specificity separately) [11]. For those with 4 or more, a bivariate model was fitted using ‘metandi’ in STATA10 [12]. The protocol specified a random-effects meta-analysis was undertaken using WinBUGS 1.4.3 [13] for tests with 3 or more risk strata, but no data were found eligible for this analysis.

Heterogeneity between study results was explored through consideration of study populations, study design, CDR and outcomes chosen, although the small number of studies in each category limited this approach. Sensitivity analysis was undertaken by comparing results when the original (derivation) data set was included and excluded.

For those areas where a quantitative synthesis was not possible, a narrative approach was used.

## Results

**Figure 1:**
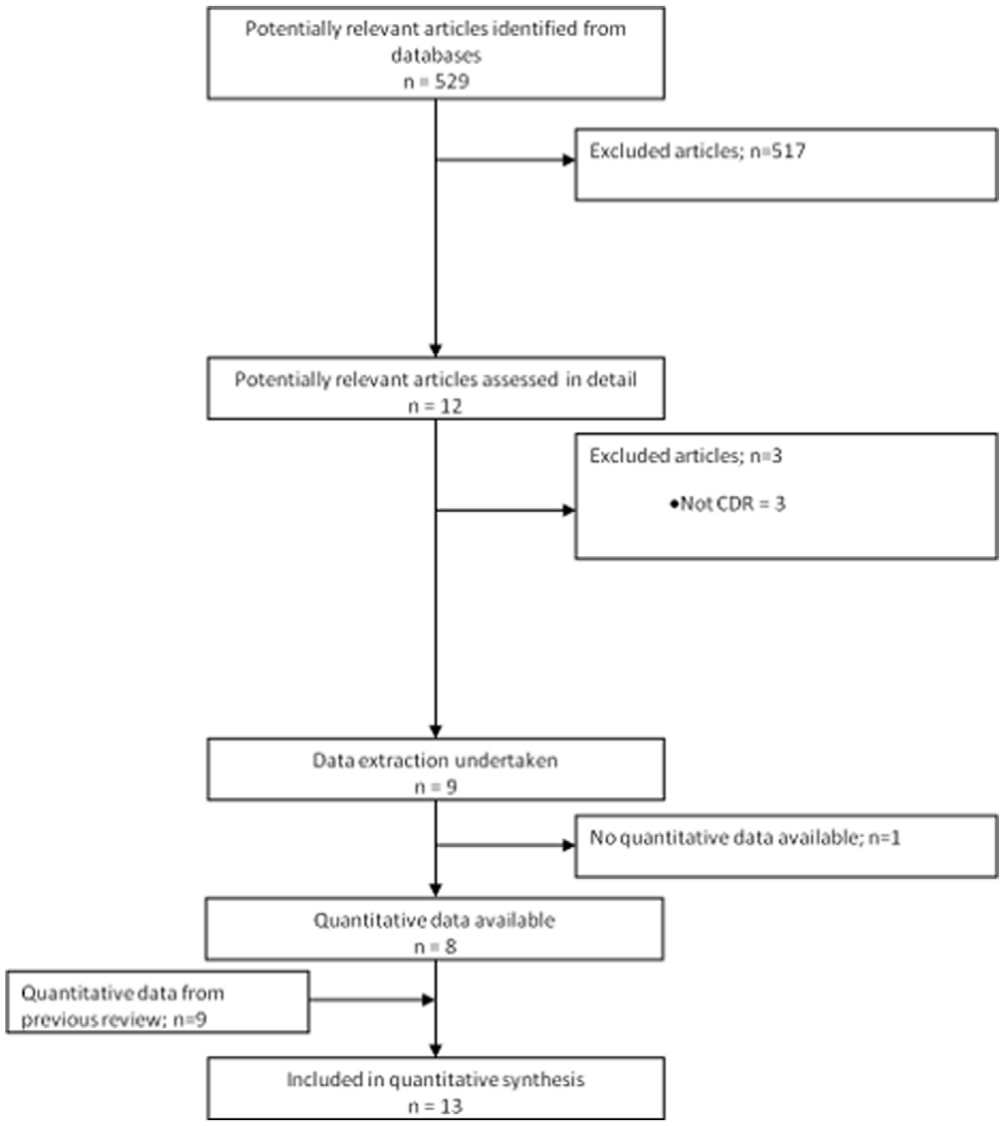
Flowchart of studies through the review.

9 articles reporting on 8 studies were eligible for inclusion in the review (see Figure 1). The studies included patients from 2 month to 22 years old, with a wide range of malignancies, and a total of 2591 episodes of FNP describing four groups of outcomes: death, critical care requirement, serious medical complication, and bacteraemia. Six studies undertook prospective data collection, two retrospective. Details of the CDR included in this review are given in Table 1.

**Table 1:** Clinical decision rules examined.

	Patient and disease related factors	Episode specific factors	Rule formulation	Outcome	Inclusion criteria	Exclusion criteria
Klaassen [24]	None	Absolute monocyte count	Absolutely monocyte count >100/mm3 = low risk of bacteraemia	Significant bacterial infection	ANC <500cells/mm^3^ or ≤1000cells/mm^3^ and falling. Temperature >38.0°C ≥2 occasions in ≥12 h, or once >38.5°C, or localised infection	New malignant diagnosis; bone marrow or stem-cell transplantation in preceding 6 months. Another medical condition that independently required inpatient observation. Interstitial infiltrate or lobar consolidation on chest x-ray
Alexander [28]	AML, Burkitts lymphoma, induction ALL, progressive disease, relapsed with marrow involvement	Hypotension, tachypnea/hypoxia <94%, new CXR changes, altered mental status, severe mucositis, vomiting or abdominal pain, focal infection, other clinical reason for in-patient treatment	Absence of any risk factor = low risk of serious medical complication	Significant adverse outcome	ANC ≤500/mm^3^, temperature >38.5°C. Outpatient status.	Post stem cell transplant
Rondinelli [27]	2 points for central venous catheter, 1 point for age >5 years	4.5 points for clinical site of infection,2.5 points for no URTI, 1 point each for fever >38.5°C, hemoglobin ≤7 g/dL	Total score <6 = low risk of serious infectious complication	Serious infectious compllications	ANC <500cells/mm^3^ or ≤1000cells/mm^3^ and falling, temperature ≥37.8°C ≥3 occasions in ≥24 h, or once >38.0°C. First episode per patient (new or relapsed disease)	Second or subsequent episode. Episodes in progressive disease (<6 m from between completing therapy and relapse). History of BMT
PINDA [26]	Relapsed leukaemia, chemotherapy within 7 days of episode	CRP ≥90 mg/dL, hypotension, platelets ≤50 G/L	Zero risk factors or only low platelets or only <7 days from chemotherapy = low risk of invasive bacterial infection	Invasive bacterial infection	ANC ≤500cells/mm^3^, axillary temperature ≥38.0°C ≥2 occasions 1 h apart, or once ≥38.5°C	Not reported
Ammann [32]	Bone marrow involvement, central venous catheter, pre-B-cell leukemia	Absence of clinical signs of viral infection, CRP >50 mg/dL, white blood cell count <0.5 G/L, hemoglobin >10 g/dL	Three or fewer risk factors = low risk of significant infection	Significant infection	Age 1–18 y, episode following non-myleoablative chemotherapy, temperature >38.5°C or >38°C for >2 h, and ANC <500 cells/mm^3^	High dose chemotherapy
SPOG (Adverse events rule) [7]	Applied after 24 hours: 4 points for chemotherapy more intensive than ALL maintenance	Applied after 24 hours: 5 points for hemoglobin >90 g/L, 3 points each for white blood cell count <0.3 G/L, platelet <50 G/L, any adverse event occurred	Total score <9 = low risk of adverse FN outcome	Significant adverse outcome	Age 1–18 y, episode following non-myleoablative chemotherapy, temperature >38.5°C or >38°C for >2 h, and ANC <500 cells/mm^3^	High dose chemotherapy
SPOG (Bacteraemia rule) [18]	None	Applied after 24 hours: shaking chills ever observed, haemoglobin >90 g/L, platelet <50 G/L, any other need for IP treatment	No risk factors = low risk of bacteraemia presenting after 24 hours	Late bacteraemia (>24 h)	Age 1–18 y, episode following non-myleoablative chemotherapy, temperature >38.5°C or >38°C for >2 h, and ANC <500 cells/mm^3^	High dose chemotherapy
Hakim [16]	Score from cancer diagnosis: AML = 20, ALL/lymphoma = 7, Solids = 0	Clinical presentation of serious unwell or toxic = 14, fever at presentation: ≥39°C = 11, ANC <100/mm3 = 10 points	Total score <24 = low risk of serious infection or sepsis	Serious infection or sepsis	Outpatient, temperature >38.3°C or >38°C for >1 h, and ANC <500 cells/mm^3^	Inpatients, stem cell transplant recipients
Hakim [16]	Score from cancer diagnosis: AML = 11, others = 0. Relapsed disease = 11. Non-white patient = 8	Clinical presentation of serious unwell or toxic = 20	Total score <20 = low risk of any medical complication	Any medical complication	Outpatient, temperature >38.3°C or >38°C for >1 h, and ANC <500 cells/mm^3^	Inpatients, stem cell transplant recipients
Delebarre [14] (abstract only)	1 point for hematological malignancy, chemotherapy at high-risk of prolonged neutropenia,	1 point for clinical signs of local infection, fever >39°C, white cell count <500/mm3 or monocytes <100/mm3 and procalcitonin >0.3 ng/ml. TWO points for severe sepsis.	High risk >1 point.	Severe infection	Unclear	Unclear
Mian [15] (abstract only)		No clear rule – uses postive blood culture result and raised CRP	No clear rule	Intensive care admission	Unclear	Unclear
Badiei [17]		Platelets <20 g/dL, temperature ≥39°C, ANC <100/mm3, mucositis, abnormal CXR on presentation	Risk of infection greater with more risk factors: no single threshold applied	Life threatening infection	Outpatient, temperature >38.5°C or >38°C for >1 h, and ANC <500 cells/mm3	BMT, Fever with new diagnosis, inpatient status

AML = acute myeloid leukaemia; ANC = Absolute neutrophil count; BMT = bone marrow transplant; CXR = chest radiograph; CRP = C-reactive protein; PINDA = Programa Infantil Nacional de Drogas Antineoplásticas; SPOG = Swiss Pediatric Oncology Group; URTI = upper respiratory tract infection.

### Quality assessment

The studies varied in quality. Potential biases due to threats to independent outcome assessment were present in two studies [2], [14], verification bias in two [2], [7], and two were presented only as abstracts [14], [15]. Five definitions of febrile neutropenia were used, with five definitions of fever and two of neutropenia. However, all definitions were clinically similar, with variation was mainly in the duration of time for a lower temperature to be considered ‘prolonged’.

### New CDR derivations

Five studies attempted to derive at least one CDR. Four studies examined rules to predict significant medical complications; a group of outcomes generally encompassing death, intensive care admission, significant bacterial or fungal infection, and need for organ support such as supplemental oxygen, inotropes or dialysis [7], [14], [16], [17]. Two examined rules to predict bacteraemia [16], [18], and one intensive care admission [15]. In one case a clear CDR could not be assessed [15]. The CDR used data from the initial/admission assessment, or from a later assessment after approximately 24 hours of observation. The new CDR generally had high sensitivity for the chosen outcome at the expense of poor specificity (see Table 2) and considered patient-disease, patient-episode and laboratory factors. Considerable imprecision in the estimates was seen, due mainly to the small numbers in individual studies (fewer than 350 patients).

**Table 2:** Diagnostic test accuracy of newly described CDR.

	Number of patients	Number of episodes	Age of patients	Outcome	Number with poor outcome	Proportion in low risk group	Sensitivity	Specificity
Hakim [16]	332	332	Median 6 yrs, range 2.4 months to 21.6 years	Serious infection or sepsis	47	69%	74.5% (95% CI 60.5% to 84.7%)	76.4% (95% CI 71.1% to 81.1%)
				Medical complications	40	63.7%	77.5% (95% CI 62.5% to 87.7%)	69.5% (95% CI 63.9% to 74.5%)
Delebarre [14](abstract only)	146	316	Mean age 8 years, range 0.5 yrs to 17.5 yrs		70	20.6%	98.6% (95% CI 92.3% to 99.7%)	26% (95% CI 20.9% to 31.8%)
Badiei [17], Threshold value: 0 risk factors	68	120	Mean 5.9 years	Life threatening infection	35	29.2%	97.1% (95% CI 85.5% to 99.5%)	40% (95% CI 30.2% to 50.6%)
1 risk factor					35	64.2%	71.4% (95% CI 54.9% to 83.7%)	78.8% (95% CI 69% to 86.2%)
2 risk factors					35	75%	62.9% (95% CI 46.3% to 76.8%)	90.6% (95% CI 82.5% to 95.2%)
3 risk factors					35	85%	40% (95% CI 25.6% to 56.4%)	95.3% (95% CI 88.5% to 98.2%)
4 risk factors					35	98.3%	5.7% (95% CI 1.6% to 18.6%)	100% (95% CI 95.7% to 100%)
SPOG [7] (Adverse events rule)	206	423	6.9 years (IQR 3.8 years to 11.6 years)	Serious adverse medical outcome	122	35%	92% (IQR 91% to 93%, range 90% to 98%)	45% (IQR, 38% to 49%, range 12% to 57%)
SPOG [18] (Bacteraemia rule)				Late bacteraemia	67	36%	93% (IQR 91% to 97%)	41% (IQR 21% to 45%)

CI = confidence intervals; IQR = interquartile range.

The newly derived CDR were subject to validation by internal statistical means (cross-validation) or in one alternative data set (see Table 3). In all except one case [15], multivariable regression analysis was used to build the model. One rule was built with a classification and regression tree (CART) approach [15].

**Table 3:** Validations of rules.

Rule	Number of patients	Number of episodes	Age of patients	Outcome	Proportion in low risk group	Sensitivity	Specificity
**Klaassen rule**							
**Klaassen** [24] (derivation)	140	227	Median 6.8 y (range 6 m to 17 y: derivation set)	Significant bacterial infection	37%	87.5% (95% CI 76.4% to 93.8%)	41.2% (95% CI 35.9% to 46.6%)
Amman [7]	206	423	6.9 years (IQR 3.8 years to 11.6 years)	Serious adverse medical outcome	38%	86.9% (95% CI 79.8% to 91.8%)	48.5% (95% CI 42.9% to 54.1%)
Macher [20]	167	377	Median 6 y, range7 m to 19 y	Significant bacterial infection	40%	79.3% (95% CI 61.6% to 90.2%)	45% (95% CI 35.9% to 54.3%)
Baorto [22]	558	1171	Mean 8.0 y (range 1 y to 23 y)	Bacteraemia	21%	88.9% (95% CI 83.6% to 92.6%)	22.4% (95% CI 19.9% to 25.1%)
Madsen [23]	76	157	Mean 8 y (range 2 m to 18 y)	Microbiologically documented infection	39%	91.7% (95% CI 74.2% to 97.7%)	41% (95% CI 35.5% to 46.8%)
Rackoff [21]	72	172	Range 9 m to 18 y: derivation set	Bacteraemia	19%	100% (95% CI 89.8% to 100%)	23.2% (95% CI 16.9% to 30.9%)
**Ammann 2003 rule**							
**Ammann** [32] (derivation)	132	364	Not reported	Severe bacterial infection	14%	100% (95% CI 95.9% to 100%)	22.7% (95% CI 16.8% to 30%)
SPOG [7]	206	423	6.9 years (IQR 3.8 years to 11.6 years)	Severe bacterial infection	10%	96.7% (95% CI 91.9% to 98.7%)	12.3% (95% CI 9.1% to 16.5%)
Macher [20]	167	377	Median 6 y, range7 m to 19 y	Severe bacterial infection	8%	95.2% (95% CI 86.9% to 98.4%)	9.1% (95% CI 6.4% to 12.8%)
**Alexander rule**							
**Alexander** [28] (derivation)	104	188	Mean 8.9 y, SD 5.7	Adverse medical complication	58%	84.6% (95% CI 57.8% to 95.7%)	64.6% (95% CI 53.8% to 74.1%)
SPOG [7]	206	423	6.9 years (IQR 3.8 years to 11.6 years)	Adverse medical complication	8%	94.3% (95% CI 88.6% to 97.2%)	8.6% (95% CI 6% to 12.4%)
Dommett [2]	368	762	Median age 5 years 7 months (range 1 month to 17 years 6 months).	Adverse medical complication	53%	58.7% (95% CI 52.2% to 65%)	57.9% (95% CI 53.7% to 62%)
**Programa Infantil Nacional de Drogas Antineoplásticas (PINDA) Rule**							
**Santolaya** (derivation) [26]	257	447	Mean 7 y (range 6 m to 18 y)	Invasive bacterial infection	43%	85.4% (95% CI 79.5% to 89.8%)	64.6% (95% CI 58.2% to 70.5%)
Santolaya (validation) [25]	170	263	Mean 7 y (range 7 m to 17 y)	Invasive bacterial infection	40%	92.1% (95% CI 86.5% to 95.6%)	76.4% (95% CI 68.2% to 83.1%)
SPOG [7]	206	423	6.9 years (IQR 3.8 years to 11.6 years)	Invasive bacterial infection	15%	93.4% (95% CI 87.6% to 96.6%)	18.9% (95% CI 14.9% to 23.7%)
Macher [20]	167	377	Median 6 y, range7 m to 19 y	Invasive bacterial infection	46%	66.7% (95% CI 51% to 79.4%)	48.1% (95% CI 41.4% to 54.8%)
**Swiss Pediatric Oncology Group (SPOG) 2010 Rule**							
**SPOG** [7] (Adverse events rule derivation)	206	423	6.9 years (IQR 3.8 years to 11.6 years)	Serious adverse medical outcome	35%	92% (IQR 91% to 93%, range 90% to 98%)	45% (IQR, 38% to 49%, range 12% to 57%)
Miedeima [19]	110	210	Median 6.6 years (IQR 4.3 years to 10.8 years)	Serious adverse medical outcome	50%	82% (95% CI, 77% to 87%	57% (95% CI, 50% to 64%)
**Rondinelli Rule ** [**27**] ** (no derivation data)**							
Macher [20]	167	377	Median 6 y, range7 m to 19 y	Severe bacterial infection	N/A	62% (36–82)	43% (34–52)
SPOG [7]	206	423	6.9 years (IQR 3.8 years to 11.6 years)	Severe bacterial infection	35%	84.4% (95% CI 77% to 89.8%)	43.2% (95% CI 37.7% to 48.8%)

### Validation of CDR

Four studies [2], [7], [19], [20] were explicit in undertaking validations of 9 previously described CDR. These universally demonstrated poorer discriminatory ability when tested in alternative data sets (see Table 3). The geographical settings for validations of the rules varied from those where the rule had been derived.

### Synthesis of CDR accuracy

Meta-analysis was undertaken for two CDR; the “Klaassen” rule and the “Ammann” rule. Two further CDR, the PINDA rule and the “Alexander” rule, have not been subject to meta-analysis as the results are too heterogeneous, these results are presented graphically. Two further CDR, the Rondellini rule and the SPOG2003 rule, have been assessed in two datasets, too few to perform meaningful meta-analysis. No data were available to update the three-stratum “Rackoff” rule meta-analysis of the previous study [3].

**Figure 2:**
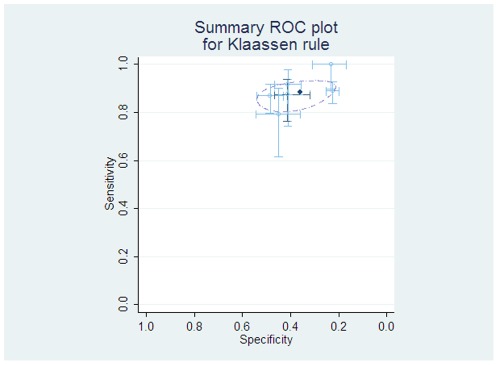
Individual and pooled diagnostic test accuracy of ‘Klaassen’ rule. The ROC space plots show each study estimates of sensitivity and specificity as a marker at the point estimate, with 95% confidence intervals demonstrated by lines. In reading such graphs, tests with a better discriminatory ability fall in the top left corner of the plot, and non-discriminatory tests fall on a 45° line between the bottom left and top right. The light lines and circles represent individual studies, with the darker dashed lines showing the study from which the rule was derived. The dark circle is the pooled estimate of sensitivity and specificity, and the dashed ellipse represents the bivariate 95% confidence intervals of this result.

The “Klaassen” rule is based on a single feature: an absolute monocyte count of greater than 100/mm^3^ to predict patients less likely to have significant infection. Data were pooled from 4 studies from the previous review [21], [22], [23], [24] and two new sources [7], [20]. The results of this analysis give a pooled average sensitivity of 88% (95% CI 84 to 91%) and specificity of 36% (95% CI 27 to 45%), see Figure 2.

**Figure 3:**
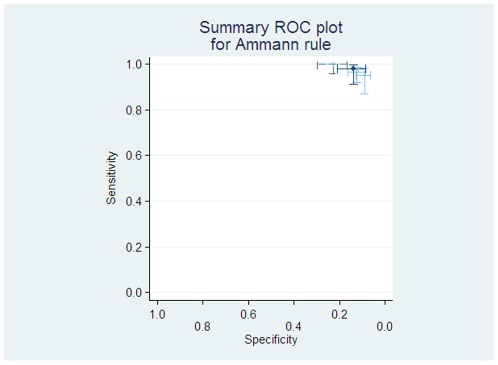
Individual and pooled diagnostic test accuracy of ‘Ammann’ rule. The ROC space plots here has the light lines and circles represent individual studies, with the darker dashed lines showing the study from which the rule was derived, and the heavy dark lines the pooled estimate of sensitivity and specificity, with the univariate 95% confidence intervals.

The “Ammann” rule describes patients at low risk of significant bacterial infection as from weighted factors including: bone marrow involvement, clinical signs of viral infection, serum C-reactive protein (CRP) level, leukocyte count, presence of a central venous catheter, high haemoglobin level, and diagnosis of pre-B-cell leukaemia (see Table 1 for details). Three studies provide data to test this rule [7], [18], [20]. The pooled average sensitivity was 98% (95%CI 91 to 99%) but pooled average specificity only 13% (95% CI 8% to 21%), see Figure 3.

**Figure 4:**
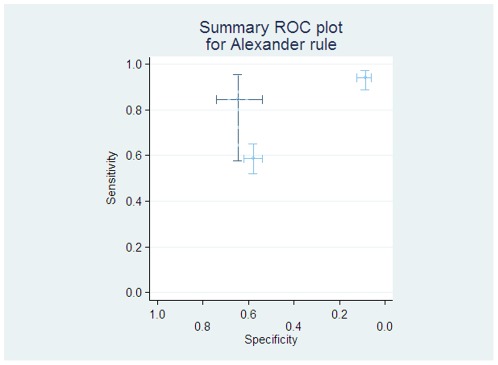
Individual diagnostic test accuracy of ‘Alexander’ rule. The light lines and circles represent individual studies, with the darker dashed lines showing the study from which the rule was derived.

The “Alexander” rule examined adverse clinical consequences, using a combination of clinical features which predict prolonged neutropenia, and significant co-morbidities at presentation. This rule was assessed by two further studies [2], [18]. There was marked heterogeneity in the results of these three studies (see Figure 4). When used at reassessment after 48 hrs of hospitalisation, there was marked improvement in the discriminatory ability of the rule [2] (sensitivity = 100%, specificity = 39%).

**Figure 5:**
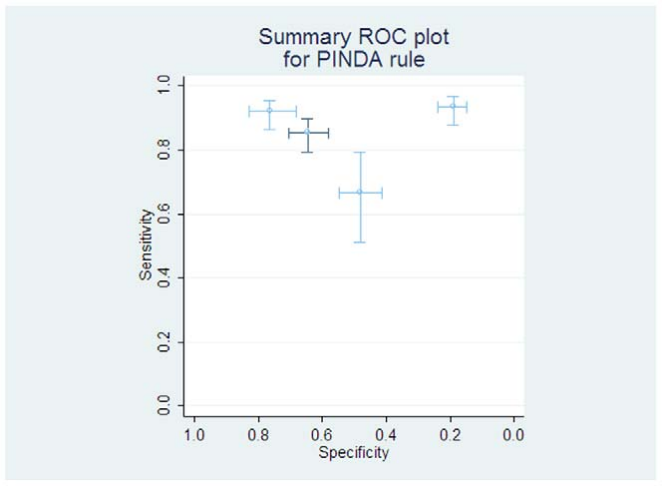
Individual and pooled diagnostic test accuracy of ‘PINDA’ rule. The light lines and circles represent individual studies, with the darker dashed lines showing the study from which the rule was derived.

The PINDA rule again describes patients at low risk of significant bacterial infection as from weighted factors including laboratory and chemotherapy related parameters. This has been examined in two studies from the Santolya group [25], [26] and by two validations from European centres [7], [20]. There was marked heterogeneity (see Figure 5), potentially explained through geographical variation: the rule worked well applied in the population in Chile, but failed to differentiate patients in French and Swiss/German studies.

The rule of Rondellini [27] is a weighted score of clinical and haematological parameters (see Table 1 for details) and was assessed in two validation datasets. These demonstrated a sensitivity of 84% [7] and 62% [20] and both estimated specificity at 43%.

The SPOG2003 is a weighted score of haematological parameters with intensity of chemotherapy. It is applied after 8–24 hours of hospitalisation. This model was shown to have a sensitivity 92% and specificity of 45% [7]. A validation of this model demonstrated poorer sensitivity (82%) and slightly better specificity (57%) [19].

## Discussion

This update systematic review builds on previous work to bring our knowledge of currently developed clinical decision rules for risk stratification in paediatric febrile neutropenia up to date. Now nine further models have been described, bringing the total to 25, and have included 10,000 episodes. It remains the case that no one rule is clearly better than any other, but we are now more clearly aware of the limitations of CDR which have not been subject to temporal and geographical validation.

The majority of CDR in this review focus upon defining a group at ‘low risk’ of complications. These rules once again have clinical and physiological similarities. The dominant themes are of a relationship between underlying diagnosis, chemotherapeutic regime, and clinical and laboratory parameters at the outset of the episode of fever. A further finding from this review is the demonstration that undertaking risk stratification at 24–48 hours after the onset of the episode leads to much greater discrimination, as many occult infections will have declared in this period.

Two rules have shown relative consistency of results. These are the simplest stratification of patients using the criteria of absolute monocyte count >100/mm^3^ to define a low risk group [24]. This has a pooled average sensitivity of 88% (95% CI 84 to 91%) and specificity of 36% (95% CI 27 to 45%), and if we assume serious infectious events occur in 30% of the group, the low-risk group has a 9% risk of serious infection, and accounts for approximately 29% of the total population. The high risk group has a 37% risk of infectious complications.

The Ammann 2003 rule [7] has much better sensitivity (estimated at 98%), leading to a risk of serious infectious complications in around 5% cases, but would only class 9% of patients as low risk, making it of little practical use.

Other further rules have shown marked heterogeneity: the Alexander rule [28] and the PINDA rule. The data support the use of the PINDA rule in Chile, where it has been successfully validated [25], but do not support its use in Europe. A similar situation exists with the Brazilian rule [27] which again was not successfully validated in European data sets. The Alexander rule did not successfully differentiate patients at admission in the UK and Europe, but its use at a 48 hour reassessment was associated with successful reductions in hospital stay. A further, newer, rule from the SPROG group requires more validation before a decision can be made on its usefulness.

These findings, that validation of CDR may be poor in comparison to derivation, and that geographical variation may mean CDR fail to work universally, have important clinical implications. There is a wealth of examples in the statistical and methodological literature regarding the over-optimism of newly derived CDR [29], [30]. The core concept is that rules derived from one dataset fit the idiosyncrasies and anomalies of the data collected, rather than reflecting the predictive power in the whole population of children experiencing FNP. However, these frequently equation-laden papers are uncommonly read by clinicians, and the complex approaches suggested to ‘shrinking’ the CDR values to increase their reproducibility are tricky to understand and to implement. The finding of geographical variation is potentially through different interpretations of similar findings; for example, how “unwell” should a child appear before they fall into this diagnostic category? There may also be subtle differences in the regimes used, as an example the use of steroid pulses in maintenance treatment for acute lymphoblastic leukaemia varies across Europe, and this may affect the CDR discriminatory ability.

This review has demonstrated there is an increasingly wide range of rules mainly for the prediction of an absence of adverse outcomes during episodes of febrile neutropenia in children, despite the existence of at least sixteen other applicable CDR [3]. Six rules have been subject to further verification, each demonstrating a variable degree of over-optimism in the original reports when the CDR is applied in different settings. The small size of these reports, with low ratios of events per variable examined may explain some of the variability in factors selected and poor reproducibility, as may undefined aspects of geographical differences between populations.

The practical application of these CDR requires it to be appropriate to the healthcare setting, and validated in the setting in which it is to be used. There remains a need for further research to reduce uncertainty around the efficiency of CDR, and potentially generate a very robust model on the basis of a much larger dataset, with well over 20 events per variable under examination. Importantly, rules should also identify a group at the highest risks of complications, to concentrate hopefully lifesaving early sepsis interventions in this group [31]. This project is already underway, with the PICNICC collaboration having collected data on around 5000 episodes of febrile neutropenia from 18 collaborating groups across North & South America, Europe and Asia.
